# Salivary proteins and microbiota as biomarkers for early childhood caries risk assessment

**DOI:** 10.1038/ijos.2017.35

**Published:** 2017-11-10

**Authors:** Abdullah S Hemadi, Ruijie Huang, Yuan Zhou, Jing Zou

**Affiliations:** 1 State Key Laboratory of Oral Diseases & National Clinical Research Center for Oral Diseases & Department of Pediatric Dentistry, West China Hospital of Stomatology, Sichuan University, Chengdu, China

**Keywords:** caries risk assessment, early childhood caries, salivary microorganisms, salivary proteins

## Abstract

Early childhood caries (ECC) is a term used to describe dental caries in children aged 6 years or younger. Oral streptococci, such as *Streptococcus mutans and Streptococcus sorbrinus*, are considered to be the main etiological agents of tooth decay in children. Other bacteria, such as *Prevotella* spp. and *Lactobacillus* spp., and fungus, that is, *Candida albicans*, are related to the development and progression of ECC. Biomolecules in saliva, mainly proteins, affect the survival of oral microorganisms by multiple innate defensive mechanisms, thus modulating the oral microflora. Therefore, the protein composition of saliva can be a sensitive indicator for dental health. Resistance or susceptibility to caries may be significantly correlated with alterations in salivary protein components. Some oral microorganisms and saliva proteins may serve as useful biomarkers in predicting the risk and prognosis of caries. Current research has generated abundant information that contributes to a better understanding of the roles of microorganisms and salivary proteins in ECC occurrence and prevention. This review summarizes the microorganisms that cause caries and tooth-protective salivary proteins with their potential as functional biomarkers for ECC risk assessment. The identification of biomarkers for children at high risk of ECC is not only critical for early diagnosis but also important for preventing and treating the disease.

## Introduction

Dental caries are one of the most common chronic infectious diseases of preschool-aged children, characterized by the destruction of tooth tissues by synergistic complex effects among acids generated from the fermentation of dietary carbohydrates by bacteria and susceptible host factors, such as teeth and saliva.^1^ Tooth decay of primary teeth in children 71 months of age or younger is referred to as early childhood caries (ECC) and affects 23% of preschoolers in the USA and over 60% of children in China.^2, 3^ Any sign of smooth-surface caries in children younger than 3 years of age is indicative of severe ECC (S-ECC).^4^ S-ECC is indicated by children from ages 3 through 5 years who have one or more cavitated missing teeth (due to caries), filled smooth surfaces in primary maxillary anterior teeth, or decayed, missing, or filled score of ≥4 (age 3 years), ≥5 (age 4 years), or ≥6 (age 5 years) surfaces.^4^ Destruction of primary teeth has already occurred when ECC is present, which is not only harmful to a child’s physical health but also has psychological and emotional effects.^5^ Thus, the preventive intervention of and early diagnosis of ECC are of particular clinical importance. Recent studies have focused on the assessment of risk factors and oral defense mechanisms in preventing ECC. We used the PubMed, EMBASE, Medline and OVID databases to search for related keywords to outline recent advances.

Many studies have correlated mutans streptococci with ECC.^6, 7, 8^ A systematic review by Parisotto *et al.*^8^ found that the count of salivary mutans streptococci is a strong risk indicator for ECC. Vachirarojpisan *et al.*^9^ noticed that the mutans streptococci level in unstimulated saliva was a statistically significant indicator of ECC, with an odds ratio (OR)=4.5; 95% confidence interval (CI):  1.8–11.7. A correlation between lactobacilli and caries increment was also found in young children (3–4 years of age), with an OR=16.2; 95% CI: 1.12–233.36,^10^ as well as a relative risk=2.70; 95% CI: 2.23–2.99.^11^ In addition to mutans streptococci and lactobacilli, *Candida* spp. is frequently present in the oral cavity of children with ECC.^12, 13^

Saliva is a complex body fluid composed of organic and inorganic constituents that are essential for the health of the oral cavity. Saliva mainly originates from three pairs of major salivary glands, that is, parotid glands, submandibular and sublingual glands, and from numerous minor salivary glands situated in the oral submucosa.^14^ In addition to inevitable mixing with gingival crevicular fluid, saliva also contains desquamated cells of the oral epithelium, microorganisms, bronchial expectoration remains and food debris.^15^ Saliva principally consists of 99.5% water, 0.3% proteins and 0.2% trace and inorganic substances.^16^ Saliva is one of the innate defense systems of the human body that protects teeth by several mechanisms, such as improving tooth enamel by remineralization, neutralizing low plaque pH, rinsing food debris, microorganisms and sugar aggregation, and by its antibacterial and bacterial properties.^17, 18^ The concentration of proteins and polypeptides present in saliva is important in the maintenance of oral health and homeostasis, as increased frequency and severity of oral disease are often associated with qualitative and quantitative changes of the saliva proteome.^19, 20^ Proteomic molecules, such as histatins, mucin, lactoperoxidase, defensins, proline-rich peptides and lactoferrin (LF) regulate the microbial flora of the oral cavity by exerting direct antibacterial effects.^21, 22^ Many of the proteins present in saliva are critical for the protection of oral tissues against fungal or viral infections.^23^ Therefore, salivary protein composition may play an important role in the etiology of oral disease prevalence and dental caries development.^21^ Saliva collection and storage is easy, non-invasive, relatively inexpensive and is low risk for both the patient and medical staff. These characteristics of saliva are advantageous when studying caries biomarkers in infants, children and adults. In recent studies, saliva was used to evaluate the incidence of caries by examining bacterial abundance, protein identity and concentration, and buffer capacity within the saliva samples.

The physiological states of the human body can be examined by monitoring changes in the composition of saliva.^21, 24, 25^ The changes in salivary protein composition with aging were significantly correlated with dental caries prevalence.^26^ Saliva secretion and concentration differs depending on age and gender.^27^ The differences in total protein concentration of human whole saliva varies among different ages groups, as salivary protein concentration increases with increasing age.^28^ Changes in saliva production with aging are correlated with higher caries risk and may be an index of caries incidence.^29^ In a longitudinal study, salivary concentrations of alpha-defensins 1–3, cathelicidins LL-37, LF, calprotectin and salivary immunoglobulin A (IgA) in 1-year-old children were measured at baseline. The concentrations of the same salivary components were measured in the same children at 3 years of age. The results showed increased concentrations of antimicrobial peptides (AMPs), IgA and *Streptococcus mutans* over time.^30^

This review aims to summarize the following: (1) whether salivary microbes can be utilized as biomarkers for ECC risk assessment, (2) the influence of salivary proteins on oral microorganisms and caries occurrence and (3) whether salivary proteins can be used as biomarkers to predict ECC susceptibility and outcomes.

## Microorganisms as a risk factor for ecc

### Mutans streptococci and lactobacilli

Dental caries is the most common chronic infectious oral disease among children and teenagers in the world. Without early prevention, this infectious disease can lead to destruction of dental hard tissue and, eventually, tooth loss. A group of cariogenic bacteria, particularly mutant streptococci, *S. mutans* and *Streptococcus* *sobrinus*, are the species most frequently associated with tooth decay in children.^31, 32^ Mutans streptococci are particularly cariogenic because of their aciduric and acidogenic properties and their ability to produce intracellular and extracellular polysaccharides that facilitate bacterial attachment on the tooth surface. Many studies have demonstrated a significant correlation between high caries prevalence in preschool children and a higher mutans streptococci and lactobacilli load in saliva or plaque.^6, 8, 33, 34, 35, 36, 37, 38, 39, 40, 41^ A systemic review provided by Thenisch *et al.*^42^ discussed the correlation between salivary mutans streptococci and ECC. The group found that the presence of mutans streptococci in the saliva of preschool children without caries is associated with considerably increased caries.^42^ It has been observed in studies that measured the mutans streptococci counts in saliva (4 reports; a total of 451 children) that the pooled risk ratio of caries was 2.11; 95% CI: 1.47–3.02. A longitudinal study that followed 39 children from the age of 2 years to the age of 4 years determined that the timing of colonization may impact the severity of disease. If colonization of *S. mutans* occurred at 2 years of age, the caries index values of decayed, missing and filled teeth (dmft) were higher (dmft=10.6±5.3) at the age of 4 years than if children were colonized after the age of 2 years.^43^ By contrast, some studies have reported no significant association between ECC in multivariate regression or high levels of mutans streptococci in saliva.^7, 44^ The lack of association between the salivary bacterial level and ECC incidence may be due to increased localized bacterial adherence to the tooth surface. Nevertheless, as the majority of studies support a positive correlation between salivary levels of mutans streptococci and ECC, mutans streptococci could serve as a marker for ECC onset. In addition to mutans streptococci, lactobacilli play a role in the progression of caries.^31^ Lactobacillus spp. play a role in caries progression through the production of water-insoluble polysaccharides that promote bacterial attachment to the tooth surface and to other bacteria, fill the gaps between bacteria, facilitate plaque accumulation and the retention of bacteria on the tooth surface, and confine organic acids that alter the microenvironment to enrich the aciduric microflora.^45^ Although lactobacilli play a significant role in the prognosis of dental caries, it is unlikely that they play any significant role in the inception of dental caries.^46^ Therefore, salivary lactobacilli counts could be indirectly related to caries progression.

### *Candida* species

The *Candida* species of fungus asymptomatically colonizes the oral cavity together with other opportunistic pathogens. Depending on the oral and systemic conditions, these microorganisms in the mouth may shift from commensal to pathogenic microorganisms. *Candida* species are frequently detected in the oral cavity of children with ECC.^13^ *Candida albicans* invade the dentinal tubules and secrete acids promoting enamel demineralization. This fungus possesses the ability to adhere to the hydroxyapatite (HAP) substrates and dissolve HAP crystals by releasing calcium ions. Thus, *C. albicans* is a relevant pathogen in caries progression. Several reports have described the implication of *Candida* spp. in ECC and S-ECC.^12, 13, 47, 48, 49, 50, 51^ *C. albicans* can tolerate high acidic environments and produce high levels of organic acids, mainly acetic acid and pyruvic acid,^52^ which are more efficient in decreasing the pH of the milieu than the *S. mutans* secreted lactic acid. Some studies did not find a strong association between *Candida* spp. in saliva and caries levels in children with ECC.^53^ This does not diminish the potential for salivary *C. albicans* counts as a risk predictor for ECC.

### Other microorganisms

In addition to oral streptococci and *Candida* spp., other oral microbial species have been associated with ECC, such as *Biofidobacteria* spp., *Actinomyces* spp., *Veillonella* spp. and *Prevotella* spp.^54, 55^ As the composition of the microbiome may predict health or disease states, it is not surprising that the oral microbiota of children with S-ECC differs significantly from that of their caries-free counterparts.^56, 57^ Several studies demonstrated that *Prevotella* spp., *Actinomyces* spp., *Veillonella Porphyromonas*, *Selenomonas* spp. and an unnamed *Bifidobacterium* species were associated with ECC.^12, 57, 58^ Tanner *et al.*^55^ found an increased prevalence of *Prevotella* spp. in S-ECC (*n*=53) compared with caries-free (*n*=32) children using microarray analysis. Ling *et al.*^59^ included 60 children with and without caries (3–6 year olds) in their study and analyzed the salivary microbiome using high-throughput barcoded pyrosequencing and PCR-denaturing gradient gel electrophoresis. They observed that the detection of salivary *Prevotella* spp. was higher in caries-active children compared with caries-free children. Another study characterized the diversity of microbial flora in the saliva of children (3–4 years old) with and without caries and found that *Prevotella salivae* was more prevalent in the caries-affected group than in the caries-free group (*P*<0.05).^38^ As the abundance of *Prevotella* spp. differentiated caries-active subjects from healthy individuals, its prevalence may indicate a predictive role in the onset of dental caries. In a different study, “Microbial Indicators of Caries” was used to diagnose and predict the onset of caries over the course of 2 years in preschoolers who were clinically considered healthy. The accuracy of this prediction method and of the microbiota is similar, supporting the use of the *Prevotella* spp. for the timely prediction of caries.^60^ Hence, salivary *Prevotella* spp. may possibly be considered a predictor of ECC.

## Salivary proteins as a protective factor for ecc

Saliva is a significant factor in the development of dental caries.^61^ Saliva protects the tooth against loss of calcium and phosphate ions from the enamel by forming a dental pellicle. The salivary pellicle acts as a protective barrier and aids in preventing demineralization, promoting remineralization, keeping the oral cavity pH neutral and cleaning tooth surfaces by washing away residual food.^62^ The development of caries is influenced by the physiochemical properties of saliva, such as pH, salivary flow rate, buffering capacity, varying protein concentrations and other components of saliva. Saliva contains many innate defense molecules that participate in the protection of oral tissues by either direct antimicrobial effect or interference with microbial colonization. These molecules include AMPs (cathelicidin peptide LL-37, alpha-defensins, beta-defensins, histatins and statherin), major salivary glycoproteins (mucins, proline-rich proteins (PRPs) and immunoglobulins) and minor salivary glycoproteins (agglutinin, LF, cystatins and lysozyme). These proteins play specific functional roles in the first line of defense of the oral cavity.

### Antimicrobial peptides

AMPs are essential components of innate immunity, providing the first line of defense against oral microbial colonization and infection.^63^ Most AMPs have antimicrobial activity against Gram-negative and Gram-positive bacteria,^63^ fungi^64^ and viruses.^65^ AMPs are classified based on amino-acid composition, conformational structure and size.^66^ The most common AMPs expressed in the saliva are listed in Table 1 and described below.

#### Cathelicidin peptide LL-37

Cathelicidins are antimicrobial host defense peptides. The 18-kDa cationic alpha-helical peptide, LL-37, is found in neutrophils and inflamed epithelia, as well as in saliva.^67^ The antibacterial effect of LL-37 and its derivatives is based on their cationic property. These molecules aggregate on microbial membranes to form ion channels and transmembrane pores to ultimately cause membrane leakage and membrane rupture.^63^ In a longitudinal study, 57 toddlers (12 to 24 months old) were followed for 2–3 years. The study showed a positive correlation between elevated concentrations of salivary LL-37 (*r*=0.336, *P*<0.05) and higher numbers of *S. mutans*.^30^ This result suggests that increases in the concentration of salivary LL-37 may be a response to higher bacterial colonization. This report contradicts another study in which no correlation was found between LL-37 and mutans streptococci levels.^74^ Indeed, some reports suggest that the concentrations of salivary LL-37 in ECC have a weak or no positive correlation with dental caries.^74, 75^ Davidopoulou *et al.*^76^ found that the concentration of LL-37 was lower in children with high caries activity in the primary dentition compared with caries-free children. Therefore, it is possible that LL-37 is an important innate immunity factor in the oral cavity, playing a protective role against caries. The relationship between salivary LL-37 and ECC is still unclear.

#### Defensins

The most prominent mammalian AMPs are the defensins. Defensins are typically peptides that are 29–35 amino acids in length, antimicrobial (prototype) cations and low in molecular weight (4–5 kDa). Depending on the pattern of cysteine pairing, two types of defensins are recognized, namely, the α-defensins and the β-defensins.^77^ In *in vitro* studies, both α-defensins and β-defensins exhibit non-specific antimicrobial activity against Gram-negative bacteria, Gram-positive bacteria and *C. albicans.*^78, 79^ Ribeiro *et al.*^75^ examined salivary peptide profiles in 10- to 71-month-old children with and without ECC. The presence of α-defensins-3 and β-defensin-3 reduced the incidence of ECC. However, a different study using enzyme-linked immunosorbent assay (ELISA) showed no significant differences between α-defensins 1–3 salivary levels in 3–5-year-old children (*P*=0.06) and caries severity.^80^ Malcolm *et al.*^30^ found that higher numbers of total plaque bacteria in 3-year-old children was positively correlated with the concentration of salivary α-defensins 1–3 (*r*=0.412, *P*<0.01), suggesting a local host reaction to ECC. In one report, the salivary concentrations of human β-defensin-2 and human β-defensin-3 in children between 36 and 60 months showed no correlation with ECC.^74^ By contrast, another study of 82 pediatric patients showed a significant increase in the concentration of β-defensin-2 (2.29±0.05) ng·mL^−1^ in the ECC group compared with the caries-free group (2.15±0.07) ng·mL^−1^.^81^ The high salivary concentration of β-defensin-2 in caries-active subjects suggests that β-defensin-2 may contribute to caries susceptibility and can potentially be utilized to perform caries risk assessments in children. There have been relatively few studies assessing the relationship between salivary defensins and their usage as biomarkers for caries risk prediction for ECC; therefore, we cannot consider it as a caries risk prediction tool for ECC.

#### Histatins

Histatins are small cationic peptides consisting of at least 12 histidine-rich basic peptides, characterized by their histidine-rich structures ranging in size from 7 to 38 amino acids.^82^ The most common forms of the histidine-rich peptides are histatin 1, histatin 3 and histatin 5, with 38, 32 and 24 amino-acid residues, respectively. These three forms account for ~85% of the total histatin proteins in saliva.^83^ Histatins, especially histatin 1, may play a role in reducing bacterial colonization on tooth surfaces because it has the ability to incorporate into the acquired pellicle and block the binding site of bacteria on tooth surfaces.^84, 85^ In an *in vitro* study, histatin 1 reduced *S. mutans* adhesion onto HAP surfaces by inhibiting the adsorption of salivary high-molecular-weight glycoproteins.^85^ In a study following 106 children (10–71 months old), there was no significant association between salivary histatin 3 and ECC.^75^ In a more recent study, the salivary peptides of children with S-ECC were assessed at different time periods, mainly before treatment, 1 week and 4 weeks after treatment.^86^ Utilizing magnetic bead-based matrix-assisted laser desorption/ionization time-of-flight mass spectrometry and western blotting, the results showed that salivary histatin 1 was expressed more in children with S-ECC 4 weeks after the treatment compared with before the treatment (*P*<0.01). In one report, the level of histatin 5 in children’s saliva had no association with ECC.^74^ Another group demonstrated a significant increase in the concentration of salivary histatin 5 in children with ECC (*n*=41 average age=(5±2.3) years) in comparison with the control group (*n*=41 average age=(5±1.5) years).^81^ The most recent longitudinal study, published by Ao *et al.*,^87^ found that increased histatin peptide in the saliva correlates with increased ECC incidence. Because of the available evidence of correlation between histatin concentration and ECC, salivary histatins may be considered to increase caries risk in young children.

#### Statherin

Statherin is a low-molecular-weight (5.4 kDa) acidic protein composed of 43 amino acids. Statherin has various functions, including binding to HAP, inhibiting the spontaneous precipitation of calcium and phosphate salts from the supersaturated saliva, and inhibiting HAP crystal growth. Statherin has been suggested as a potential precursor of the acquired pellicle because it has a strong affinity to HAP. Maintaining saliva supersaturated with calcium phosphate salts is achieved by statherin, which enhances enamel remineralization, thereby maintaining the integrity of the tooth and inhibiting caries progression.^88^ Statherin reduces bacterial and fungal colonization by aggregating the microbes. This clumping process reduces the ability of bacteria to adhere to intraoral hard and soft tissue surfaces.^88^ Shimotoyodome *et al.*^85^ found that statherin reduces *S. mutans* adhesion to HAP. Vitorino *et al.*^89^ reported a strong correlation between high levels of statherin and the absence of dental caries in children. This finding implies a protective role of statherin against caries. So far, no study has proved any association between statherin and ECC. Therefore, additional studies should be performed to further support the hypothesis that statherin can be used for caries risk prediction in ECC.

### Salivary glycoproteins

The salivary glycoproteins are a wide range of structurally and functionally composite molecules of the saliva contributing to the formation of the salivary pellicle, which influences the pattern of microbial colonization and serves as the first line of defense in the oral cavity. These glycoproteins serve as primary nutrients for the resident oral microorganisms. Two forms of glycoproteins are present in the saliva, major salivary glycoproteins (Table 2) and minor salivary glycoproteins (Table 3).

#### Major salivary glycoproteins

*Mucins*: The mucin family consists of two predominant forms in human saliva, the high-molecular-weight mucins (MG1), with molecular weights above 1 000 kDa, and the low-molecular-weight mucins (MG2), with molecular weights from 150–200 kDa. Mucins found in human saliva protect teeth from demineralization induced by the acid produced from microbial metabolism.^97^ The acquired enamel pellicle is composed primarily of MG1, which serves as an attachment site for bacteria, as well as a permeable barrier for organic acid challenge and may aid bacterial colonization on oral surfaces.^97^ Many studies have reported that MG2 can interact with oral microorganisms by promoting their agglutination.^90, 91^ Thus, MG2 may be an important antimicrobial agent. Levine *et al.*^98^ reported that salivary mucins agglutinated *S. mutans* and *S. sanguis*.^98^ In a study conducted on 120 children with and without caries, which included preschool level (aged between 4 and 6 years old, *n*=60) and school level (aged between 9 and 11 years old, *n*=60) children, the salivary mucins levels were measured using ELISA. The study reported no significant correlation between salivary mucin levels (MUC5B and MUC7) and ECC. In school children, they observed a negative correlation between salivary MUC5B and the number of decayed teeth in mixed dentition.^99, 100^ The association between salivary mucins and dental caries may be affected by changes in the oral environment from primary to permanent dentition. There is still insufficient scientific evidence supporting the correlation between salivary mucins and ECC; thus, we cannot conclude that mucins are good indicators for predicting ECC risks.

*Proline-rich proteins*: Human salivary PRPs are composed of heterogeneous molecules, which are abundant in saliva, and mainly divided into the following three groups: basic PRP (bPRP), acidic PRPs (aPRP) and glycosylated PRPs.^69^ The aPRPs play a role in the formation of the acquired dental pellicle and promote the adhesion of *S. mutans* on HAP surfaces.^84, 101^ aPRPs are important in maintaining a supersaturation of calcium ions in relation to ionic phosphate in saliva. Thus, the aPRPs may be a natural substance in preserving the calcium homeostasis of saliva.^97^ Furthermore, aPRPs play a significant role in the protection of tooth surfaces by regulating the precipitation of calcium phosphate onto dental enamel.^102^ A report by Ribeiro *et al.*^75^ identified the molecular masses of saliva from children with and without ECC (*n*=106) and concluded that the presence of proline-rich peptides (in particular, IB-4) significantly increased the chance of caries (*P*=0.035). Supporting this idea, a study by Bhalla *et al.*^99^ showed that a higher number of PRP bands were observed in the saliva of subjects with ECC. The majority of studies support the correlation between elevated PRP levels and the increased incidence of ECC, thereby indicating the potential use of PRPs as predictors for the prognosis of ECC.

*Immunoglobulins*: In addition to AMPs in the oral cavity, human saliva contains several immunoglobulins. Salivary immunoglobulins compose ~5%–15% of whole salivary proteins.^21^ IgA is the major subclass of salivary immunoglobulins (50%–60%) found in the saliva, and the rest belong to the IgG and IgM subclass. Salivary immunoglobulins are mucosal antibodies that act as the first line of defense, and they include two major antibodies, namely, secretory IgA (sIgA) and IgG.^103^ Salivary IgA constitutes 60% of the immunoglobulin in the saliva. IgA scan neutralize bacterial toxins and enzymes, interfere with the adherence of the bacteria to the tooth surface by physically blocking bacterial adhesions, inhibiting bacterial metabolism, reducing the hydrophobicity of bacteria and aggregating or clumping the bacteria together, which aids in the antibacterial action of the saliva.^21, 104^ Controversy remains in the field regarding the relationship between salivary immunoglobulin and dental caries. According to a systemic review and meta-analysis, the literature shows an association between elevated levels of salivary IgA and increased caries activity in subjects.^105^ Indeed, many studies have reported significantly higher levels of salivary IgA in children with ECC potentially pointing to sIgA as a protective mechanism against cariogenic bacteria, especially *S. mutans*.^10, 37, 61, 106, 107, 108, 109, 110, 111^ In a longitudinal study conducted by Alaluusua,^112^ the salivary IgA value increased rapidly from 0.021 g·L^−1^ (log mean 1.68±0.33) to 0.052 g·L^−1^ (log mean 1.28±0.24) in children from age 1 to 2 years. After 2 years of age, the level of salivary IgA remained constant, although the caries-active group had a significantly higher level of IgA compared with those of the caries-free group.^112^ The high concentration of salivary immunoglobulin in children with ECC may be associated with an increased antigenic load, leading to the high production of antibodies. On the other hand, one study reported an inverse relationship between salivary IgA level and caries experience in 45 3- to 6-year-old children,^113^ while another report did not observe any correlation between IgA concentration and caries activity among children aged 3–6 years.^114^ However, most studies provided evidence of higher IgA levels in children with ECC and suggest its likelihood as a caries biomarker. Salivary IgG plays a protective role in the oral cavity by inhibiting *S. mutans* growth, adherence and acid production.^115^ A cross-sectional study (*n*=90) shows that the concentration of salivary IgG was significantly higher among children with ECC (*P*<0.05).^107^ Another study supports a correlation between higher levels of total salivary IgG and ECC prevalence, whereas the total IgM level was similar in children with and without ECC.^106^ Increased production of antibodies is linked to antigenic load; hence, it is unsurprising that there are increased concentrations of salivary immunoglobulins in children with ECC. Owing to the association between ECC and increased concentrations of salivary IgA and IgG, it may be useful to use these molecules as caries risk biomarkers.

#### Minor salivary glycoproteins

*Agglutinin*: Salivary agglutinin (SAG) is a high-molecular-mass (~340 kDa) component of human saliva. SAG was originally characterized as an *S. mutans* agglutinating glycoprotein.^116^ SAG is highly glycosylated and extremely sticky, potentially binding to the pellicle and interacting with unattached bacteria, resulting in the aggregation of bacteria that are more easily swallowed or flushed away.^93, 117, 118^ Thus, SAG plays a role in the oral clearance of bacteria. Some studies have reported a correlation between increased levels of agglutinin in saliva and increased numbers of *S. mutans* in dental plaque and susceptibility to dental caries.^119^ Saliva-mediated aggregation and adherence plays a direct role in caries resistance. Caries-resistant individuals show a two-fold enhancement of saliva-mediated aggregation compared with that of the caries-susceptible group.^120^ However, owing to the lack of convincing evidence in the literature, SAG cannot yet be used as predictor for ECC incidence.

*Lactoferrin*: LF is an iron-binding cationic glycoprotein with a molecular weight of ~80 kDa. LF possesses potent activity against *S. mutans*, fungi, parasites and viruses.^121^ LF has the ability to bind and kill bacteria via direct interactions through the strongly basic N-terminal region of the glycoprotein that consists of 47 amino acids.^122^ In addition, LF and other cationic peptides are capable of neutralizing the interaction between bacterial lipopolysaccharides (LPS) and host defense cells.^123^ This interaction can alter the permeability of the outer membrane of Gram-negative bacteria and release LPS.^124^ Because of its antimicrobial activity, salivary LF is thought to play a major role in caries susceptibility. A study investigating the efficacy of toothpaste containing LF, lactoperoxidase and lysozyme found that it was capable of reducing the salivary colony forming-units of *S. mutans* and *Lactobacillus acidophilus* in children with S-ECC.^125^ In an *in vitro* study, the killing of *S. mutans* by human LF is dose-dependent.^126^ These studies suggest that LF protects against *S. mutans* colonization and can therefore be used in dental caries prevention. Another study, focusing on children (*n*=42, age range from 36 to 71 months), reported that the concentration of LF was higher in caries-free individuals than in ECC individuals. This study also found that lower concentrations of unstimulated salivary LF might have a positive relationship with ECC and may therefore be used as a predictive factor for ECC.^127^ Supporting this claim, Hao *et al.*^128^ detected higher concentrations of LF in high dmft children than in caries-free children, which may be related to caries prevention in primary dentition.^128^ However, the relationship between salivary LF and ECC is unclear. Therefore, salivary LF cannot be used as a caries risk indicator in ECC.

*Cystatins*: Cystatins belongs to a heterogeneous family of proteins with a conserved consensus sequence in their active site. Cystatins are cysteine proteinase inhibitors, antimicrobial and immunomodulatory and are present in all mucosal secretions. Cystatins have been found to bind to HAP and may therefore play an important role in the acquired dental pellicle formation, as well as a role in the enamel remineralization process.^70, 84, 95^ Moreover, higher quantities of cystatins SN and cystatin S in samples from caries-free individuals suggest that inhibition of proteolytic events on salivary proteins might indirectly provide tooth protection.^129^ At this time, it is not possible to use the cystatin level to determine the risk for ECC.

*Lysozyme*: Lysozyme is an antibacterial enzyme found in high amounts in body fluids such as saliva, serum, tears and amniotic fluid, as well as in low amounts in bile, urine and cerebrospinal fluid. Lysozyme promotes bacterial clearance through aggregation and adherence. Furthermore, it has the ability to destroy and inhibit bacterial growth.^88, 130^ In an *in vitro* study, *S. mutans* and *L. casei* were inhibited by lysozyme.^131^ A study conducted by Moslemi *et al.*^127^ examined 42 children (aged 36–71 months) and demonstrated a statistically significant increase in the salivary lysozyme level in caries-free children (9 573.81 ng·ml^−1^) compared with ECC children (2 180 ng·mL^−1^).^127^ Others have reported significantly higher concentrations of lysozyme in unstimulated and stimulated saliva in children with S-ECC compared with caries-free children.^108, 132^ The higher level of lysozyme in the S-ECC group than in the caries-free group might be due to a compensatory mechanism. In specific situations, in the presence of caries or high levels of *S. mutans*, the secretion of lysozyme, which represents a protective mechanism, may be stimulated.^127^ However, a different study has shown no significant relationship between lysozyme concentration and caries in children.^128^ Evidence that supports the relationship between lysozyme and ECC is not strong. Therefore, lysozyme cannot be used as a predictive factor for ECC incidence.

### Total salivary proteins

Several studies have investigated the relationship between the concentration of total salivary proteins and dental caries. A systemic review study conducted by Martins *et al.*^133^ reported no consistent correlation between salivary proteins and dental caries in terms of protein phenotypes, protein molecular weight or the concentration of total proteins. A recent review shows that the association between many salivary factors and dental caries has yet to be fully elucidated.^134^ Hao *et al.*^128^ reported higher levels of total salivary proteins in a caries-free group ((1 032.44±221.99) mg·L^−1^, *P*<0.001) compared with a dmft group (852.02±206.14) mg·L^−1^, suggesting an effective protective function of salivary proteins. Other studies demonstrated that the concentration of proteins was increased with caries activity in children.^135, 136^ Several other investigations did not observe a statistically significant difference in the total salivary protein concentration between children with and without ECC.^106^ It is possible that the lack of association between salivary proteins and dental caries is due to the different levels of structure and function redundancies found in saliva. The relation between dental caries in children with ECC and total salivary protein concentration is not well established. Further studies are required to ascertain the value of total salivary protein concentration as a caries risk predictor in young children.

## Conclusion

The retrieved studies show a highly significant correlation between higher caries prevalence in preschool children with higher levels of microbials, such as mutans streptococci, *C. albicans* and *Prevotella* spp., and salivary proteins, including IgA, IgG immunoglobulins, PRP and histatin peptides, in saliva compared with caries-free individuals. Therefore, based on the results of these studies, these saliva components may be used as biomarkers for ECC. To date, other salivary protein components have no association (no clear association has been found between other salivary proteins and ECC risks) with caries risk prediction, and the amount of total salivary proteinsis found not to be useful in predicting caries risk.

## Figures and Tables

**Table 1:** The most common AMPs expressed in saliva

Salivary components	Main functions	Sources	Reference
Cathelcidin LL3	Antimicrobial activity	Salivary glands and duct, neutrophil leukocytes and gingival sulcus	^67, 68^
Histatins	Antifungal and antibacterial	Parotid and submandibular salivary duct cells	^69, 70^
Alpha-defensinvHNP1–3	Microbicidal activity antiviral activity	Neutrophil granulocytes	^68, 71^
Beta-defensins hBD1 hBD2 hBD-3	Microbicidal activity and antiviral properties	Mucosal cells	^68, 71, 72^
Statherin	Remineralization of enamel	Major and minor salivary glands	^73^

**Table 2:** The most common major salivary glycoproteins expressed in saliva

Salivary components	Main functions	Sources	Reference
MUC5B (mucin MG1)	Barrier protection and lubrication	Sublingual gland	^90^
MUC7 (mucin MG2)	Agglutination of microorganisms	Submandibular and labial glands	^91, 92^
Proline-rich proteins	Remineralization of enamel	Parotid and submandibular glands	^93^
Immunoglobulins	Microbial binding; immune response	Major and minor salivary glands	^93^

**Table 3:** The most common minor salivary glycoproteins expressed in saliva

Salivary components	Main functions	Sources	Reference
Agglutinin	Antibacterial activity, aggregation of bacteria	Parotid, submandibular and sublingual glands	^94^
Lactoferrin	Antimicrobial activity	Salivary glands, mucosal epithelial cells and neutrophil granulocytes	^68, 72^
Cystatins	Protease inhibitor	Submandibular gland, parotid gland and gingival crevicular	^93, 95, 96^
Lysozyme	Antibacterial, cell wall lysis	Salivary glands, neutrophil granulocytes and gingival crevicular fluid	^68, 72^
